# The Renaissance of the Vaginal Hysterectomy—A Due Act

**DOI:** 10.3390/ijerph191811381

**Published:** 2022-09-09

**Authors:** Michael Stark, Antonio Malvasi, Ospan Mynbaev, Andrea Tinelli

**Affiliations:** 1 The New European Surgical Academy (NESA), Unter den Linden 21, 10117 Berlin, Germany; 2 Laboratory of Human Physiology, Phystech BioMed School, Faculty of Biological & Medical Physics, Moscow Institute of Physics and Technology (State University), Dolgoprudny, Moscow 141701, Russia; 3 Department of Obstetrics and Gynecology and CERICSAL (Centro di Ricerca Clinico Salentino), “Veris delli Ponti Hospital”, 73020 Scorrano, Lecce, Italy

**Keywords:** hysterectomy, vaginal hysterectomy, ten-step vaginal hysterectomy, natural orifice surgery

## Abstract

For many years, vaginal and abdominal hysterectomies were part of the routine procedures in many departments. Both of them lost their priority due to the introduction of endoscopy and robotic surgery. The disappearing abdominal hysterectomy is certainly reasonable, but the decline of using vaginal hysterectomy seems not to be justified, and it is an optimal example of the recent emergence of the Natural Orifice Surgery discipline. A modified method for vaginal hysterectomy is presented in order to encourage gynecologists to reconsider vaginal hysterectomy as a valid method. This method is the outcome of critical analyses of different vaginal hysterectomy methods. It is simple, reasonable, only ten steps, easy to learn, perform and teach, and proven to be a shorter operation with minimal blood loss and reduced need for analgesics when compared to the traditional way. Endoscopy or robotic surgery is not available everywhere. Therefore, it is important that gynecologists in low-resource settings be familiar with this simple method.

## 1. Introduction

Hysterectomy is the second most common operation performed on women after a cesarean section. Since the 19th century, all hysterectomies have been carried out abdominally or vaginally. The first abdominal hysterectomy was attributed to Charles Clay in Manchester in 1843. It was not successful, as the patient died soon after the operation. In 1853, Ellis Burnham from Massachusetts reported a successful abdominal hysterectomy, although for a wrong indication [1].

Vaginal hysterectomy was first mentioned by Soranus of Ephesus, but the first documented ones were carried out by Sauter in 1822 [2] and Recamier in 1829 [3].

The vaginal hysterectomy was, for many years, a routine surgical method. Since endoscopic surgery emerged in the 20th century, the laparoscopic hysterectomy became a popular alternative to both abdominal and vaginal hysterectomy [4] and the laparoscopic-assisted vaginal hysterectomy, which combines the vaginal approach and the endoscopic one, was popularized in Europe by Kurt Semm and by Harry Reich in the United States [5].

Today, when most gynecological operations have endoscopic solutions, the vaginal hysterectomy is gradually losing its leading role in the surgical repertoire, gradually abandoned in many centers, where trainees find the anatomical conditions complicated and develop understandable enthusiasm for endoscopy.

The vaginal hysterectomy does have major advantages when compared to abdominal or endoscopic procedures. It can be performed with epidural anesthesia, which is very important for elderly women who often belong to high-risk groups. The postoperative mobility is quick, and the need for analgesics is significantly less than in abdominal hysterectomy.

The American College of Obstetricians and Gynecologists (ACOG) recommends vaginal hysterectomy as the approach of choice whenever feasible [6].

## 2. The Surgical Method

Several vaginal hysterectomy methods were described and practiced over the years; these were the Porges, Falk, von Theobald, Heaney, Chicago, and Joel-Cohen methods. However, even in those gynecological departments where vaginal hysterectomy is still in use, different surgeons are not necessarily using the same method; therefore, there is no way to compare different surgeons and hospitals as long as no standardized methods are in use [7].

In order to standardize and simplify the vaginal hysterectomy, a critical analysis of the surgical steps used in the different methods was carried out. In the classical method, for separating the vaginal wall from the bladder in case of prolapse, an inverted “T” incision is carried out, transverse incision at the level of the internal Os followed by a median vertical incision of the vaginal wall and separating the vaginal wall laterally, opening the anterior peritoneum before handling the ligaments, grasping the sacrouterine ligaments and the paracervical tissues separately, and suturing the peritoneum. Only the common steps in all these methods were considered essential and were included, while others were abandoned. The 12 common steps were critically analyzed. Two of them seemed to have no relevance. These were the separate handling of the sacrouterine ligaments and the Paracervical tissue and suturing peritoneum.

**Figure 1:**
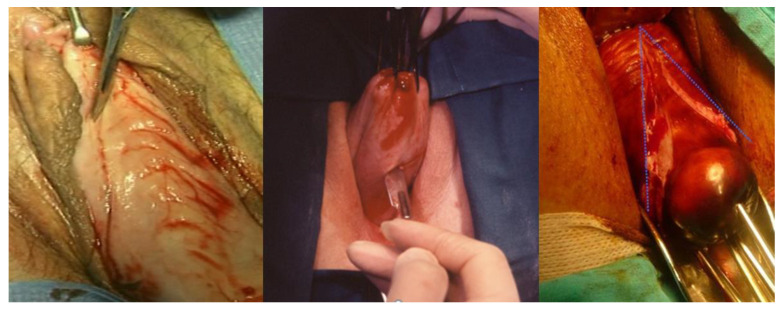
On the left, the incision on the anterior vaginal wall is shown; in the center, the incision on the posterior vaginal wall is shown; on the right, the drop-like anterior vaginal incision in women with cystocele is shown.

**Figure 2:**
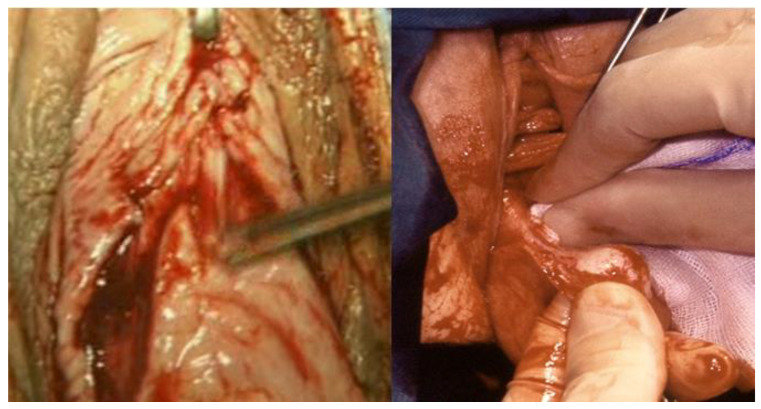
On the left, detaching the bladder away from the uterine wall; on the right, the surgeon’s hand holding the gauze separates the bladder from the uterine wall.

**Figure 3:**
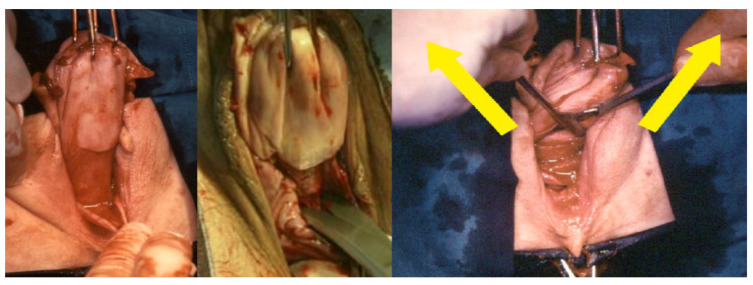
On the left, the exposure of the Douglas pouch peritoneum; in the center, the incision of the peritoneum with scissors; on the right, the scissors are pulled out while opened to expose the sacrouterine ligaments.

**Figure 4:**
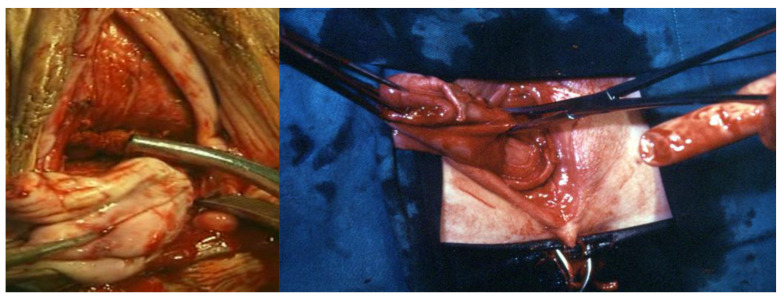
On the left, the lower blade of the Heany clamp is placed under the sacrouterine ligament; on the right, while rotating the uterus clockwise (in the left side of the uterus), the uterosacral ligaments are held together with the paracervical tissues and subsequently dissected and ligated.

**Figure 5:**
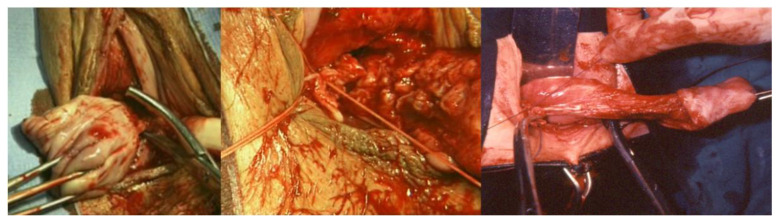
On the left, the exposure of the uterine artery; in the center, the ligation of the uterine vessels after cutting it; on the right, the dissected and tied uterine arteries.

**Figure 6:**
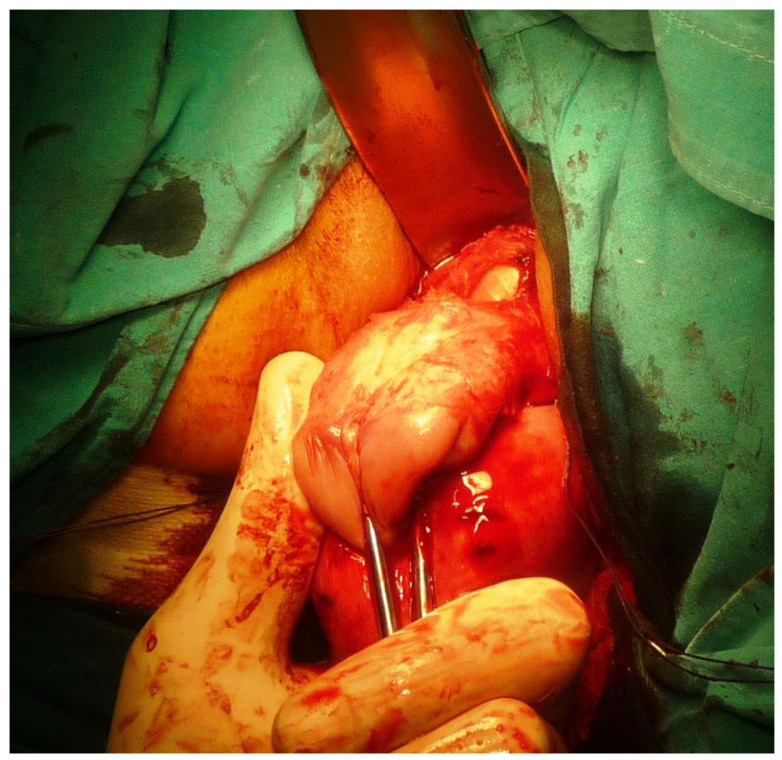
The uterus is now kept in place only by the cardinal ligaments and the utero-ovarian ligaments, so that the anterior peritoneum can be opened after lifting it with the finger, here after posterior luxation of the fundus (if the size of the uterus enables it).

**Figure 7:**
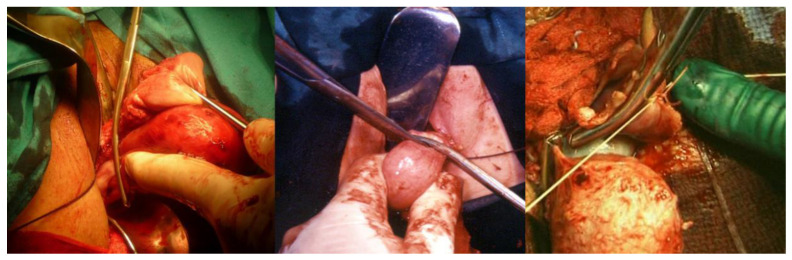
On the left, the Heaney clamp grasps the round and Utero-Ovarian ligaments and Fallopian Tubes; in the center, cutting the clamped tissues; on the right, ligation of the dissected tissues.

**Figure 8:**
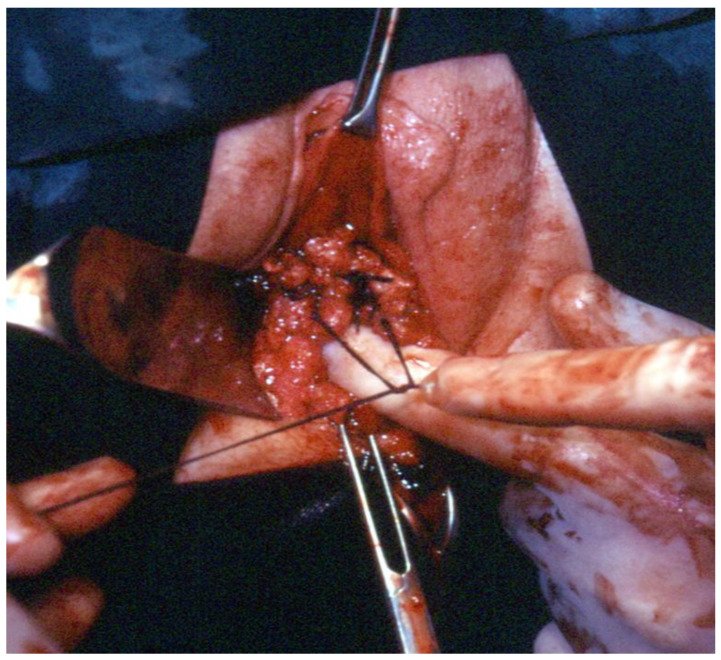
After the removal of the uterus, reconstruction of the pelvic floor, by ligating both sides, the uterosacral and the paracervical stumps.

**Figure 9:**
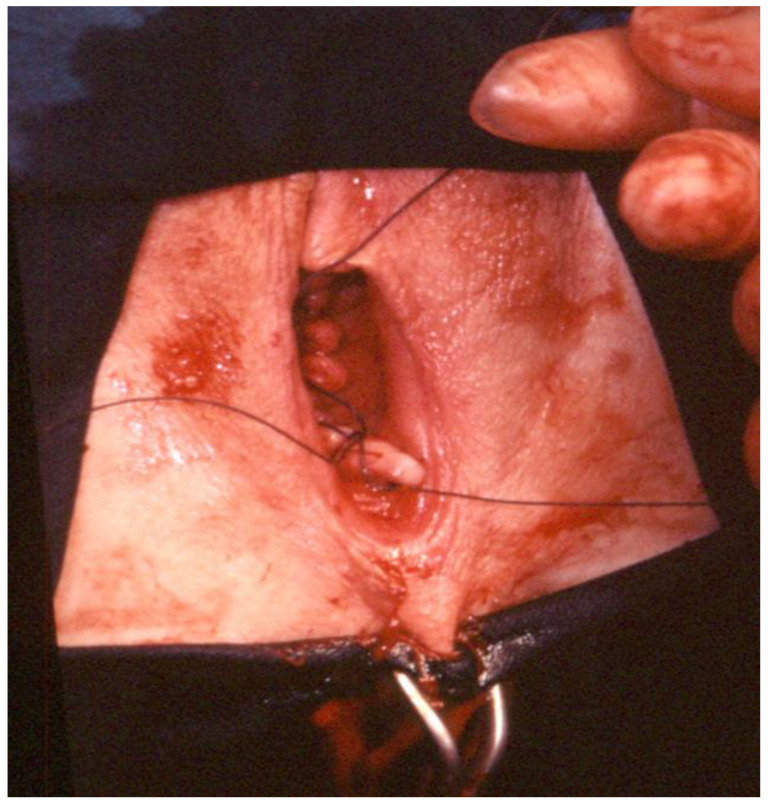
The vaginal wall is sutured continuously.

The remaining essential 10 steps are the following:

1. Circular incision of the vaginal wall where there is no prolapse and a drop-like incision starting under the urethra encircling the cervix and returning to the starting point in women with a cystocele (Figure 1). Contrary to the traditional inverted “T” incision, this step facilitates the separation of the vaginal wall from the uterus. After the drop-like incision, the tip of the drop is pulled down, separating the vaginal wall in the anatomical cleavage, usually bloodless, all the way down. At the end of this step, the cystocele repair is prepared;
2. Detaching the bladder from the uterine wall, without opening the anterior peritoneum (Figure 2);
3. Cutting the posterior peritoneum open (Figure 3);
4. Cutting and ligating the uterosacral ligaments together with the paracervical tissues (Figure 4);
5. Cutting and ligating the uterine arteries (Figure 5);
6. Opening the anterior peritoneum (Figure 6);
7. Dissection of the upper part of the uterus and appendages (Figure 7);
8. Repairing prolapse, if exists, while leaving peritoneum unsutured;
9. Reconstruction of the pelvic floor by ligating both sides sacrouterine and paracervical stumps (Figure 8);
10. Continuously suturing the vaginal wall (Figure 9).

In the first study carried out comparing the Ten-Step Vaginal Hysterectomy to the time-honored Heaney method, 44 vaginal hysterectomies using the described steps were compared to 52 vaginal hysterectomies carried out by the Heaney method. The operation time was shorter (34.1 vs. 52.3 min), and the needed time for analgesics was significantly lower (29.6 h vs. 48.7 h on average) in the Ten-Step method group [8].

The recommended suture material is PGA USP size 1 with 60 mm ½ cercle round body needle.

## 3. The Prerequisites for Vaginal Hysterectomy

It is expected that the simplification of the vaginal hysterectomy will popularize the method again, and it should be considered a favorable option.

However, despite its benefits over abdominal or laparoscopic-assisted hysterectomy, this operation needs guidance, surgical and anatomical knowledge, and accumulated experience. The performance of the different steps is based on mastering the pelvic topographical anatomy. When the vaginal hysterectomy is carried out in a woman with a partial or total prolapse, there are no difficulties in identifying the anatomical structures, such as the sacrouterine ligaments and the borders of the urine bladder. However, with no prolapse, the surgeon must rely on his/her fingertip’s sensitivity and make sure that the surgical instruments in use are placed around the right structure and in the right way. If correctly carried out, the vaginal hysterectomy should be bloodless, except for some minimal bleeding from the vaginal wall.

The needed instruments are just a few: two single-toothed tenaculum forceps, a scalpel, two Heaney clamps, two peans, surgical forceps, a needle holder, and speculums. These can be introduced to every department and certainly to countries with low resources and poor settings where endoscopy or robotic surgery is not available.

The assistant should be familiar with the surgical steps in order to be able to expose the needed areas to the surgeon. It is most important that the surgeon constantly keeps his/her eyes on the operative field and rely on the operative nurse, who should submit him/her the correct instruments in the right way. This will enable him/her to operate without losing his/her sight from the operation field.

## 4. Surgical Considerations

After pulling the cervix with two uterine tenaculum forceps, a circular incision of the vaginal wall at the level of the Internal Os is carried out. However, in the case of vaginal prolapse, a drop-like incision is made; its top under the urethra, continues parallel to the vaginal labia on the left side, its lower part posteriorly at the level of the Internal Os, and returns to the starting point parallel to the right labia. The vaginal wall should now be gently separated from the urine bladder with surgical forceps and gauze, and if carried out in the anatomical cleavage, this step is usually bloodless.

The bladder is pushed up until the anterior peritoneum is exposed, but it should not be opened during this stage because of the risk of perforating the bladder, especially in the presence of adhesions which are usually found after previous Cesarean Sections.

Instead, the posterior peritoneum is cut open first. This is carried out by lifting the cervix up, pulling the exposed peritoneum with surgical forceps, and cutting it open transversally with big curved and round-tipped scissors. The scissors are then inserted into the peritoneal cavity and are pulled back using both of the surgeon’s hands, pulling it out while extending the scissors on both handles. By doing so, the sacrouterine ligaments are exposed.

A Heaney clamp is used to hold the Sacro-Uterine ligament together with the paracervical tissue. In order to get a good hold of both structures, the lower part of the Heaney clamp is placed under the Sacro-Uterine ligament. The tenacula holding the cervix and the Heaney forceps are rotated toward each other, and after the paracervical tissue is included, the clamp is closed. Both structures are cut with scissors, so the body of the uterus is exposed, the sutures are placed above the instrument, and their full length is kept. After this stage, any uterus becomes prolapsed when pulled down, and the uterine arteries can be easily seen and palpated. After suturing and cutting them, the uterus is pulled down. The pulling of a big uterus is carried out while rotating it gently while pulling the uterus down, similar to the way vacuum delivery is carried out. The surgeon inserts his/her fingers behind the posteriorly delivered uterine fundus to lift the anterior peritoneum and open it under vision.

Occasionally, when there is a big uterus with a long parametrium, a second step should be carried out to separate the uterus from the parametrium.

The Heaney clamp is used again to separate the uterus from the round and ovarian ligaments, together with their blood vessels, which are clamped and ligated. The ligature should be placed laterally and away from the clamp, leaving the ovarian ligaments as long as possible. The uterus is cut with the scissors medial to the instruments, and a transfixion suture is placed between the clamp and the ligature, while the full length of the sutures is left long. This is carried out for security reasons because if by the traction the transfixion suture slips away, no bleeding will occur.

When removing the ovaries, the same Heaney clamp is used, and the ovarian arteries are ligated and transfixed. The bigger the uterus is, the ovarian arteries’ bow is longer. Optimally, the Heaney clamp should be placed on the ovarian arteries after the fundus is exposed. If necessary, repair of a prolapse or stress incontinence is carried out in this stage. The peritoneum is left open, and all the sutures of the sacrouterine ligaments and the round and ovarian ligaments are ligated to each other, and the vaginal wall is closed continuously.

## 5. Discussion

The “Ten-Step Vaginal Hysterectomy” is a designed operation that is the outcome of the analysis of the details of different surgical methods. Only the common steps of all of them are included as it seems that the others seem not to be essential.

Certainly, vaginal hysterectomy is a preferred method when compared to abdominal or endoscopic hysterectomy due to the lack of need for general anesthesia, the early mobility, lack of scars, and less need for analgesics.

Therefore, the question arises: Why is the vaginal hysterectomy not the routine method for any non-malignant hysterectomy?

These days, when the use of endoscopy and robotics is rising, many surgeons feel that they should use them in order to be using the current state of the art, therefore abandoning traditional methods used in the past.

The abdominal hysterectomy has not been the state of the art for years, and certainly for good reasons, due to the long hospital stay, the need for analgesics, and the remaining abdominal scar. The use of endoscopy (for a total hysterectomy, subtotal hysterectomy or laparoscopic-assisted vaginal hysterectomy) became the routine operative way. No doubt that endoscopy has many advantages over open surgery concerning hospital stay, the need for analgesics, and small abdominal scars.

Lately, a new discipline has emerged—Natural Orifice Surgery. Some procedures can already be carried out using the Douglas pouch [9,10].

The vaginal hysterectomy is certainly an optimal example of Natural Orifice Surgery which has been in use since the 19th century. Time-honored methods do not lose their value if they show advantages over other methods. Endoscopy, or robotic surgery, is not available in low-resource countries. Therefore, it is important that gynecologists in these countries be familiar with this method.

Certainly, vaginal hysterectomy needs training and demands excellent anatomical knowledge and professional integrity in order to be able to decide when to stop the procedure and convert it to an endoscopic or open procedure.

After the introduction of the Ten-Step Vaginal Hysterectomy, some groups have already started to use this method [11,12,13,14]. Without any exception, all reports have favorable outcomes when compared to the traditional vaginal hysterectomy, concerning operation time, blood loss, and the need for analgesics.

In a randomized study in Bangladesh which was carried out 6 years after the original description of the Ten-Step Vaginal Hysterectomy, 54 operated with the Heaney were compared to 56 women operated on with the Ten-Step Vaginal Hysterectomy. There was significantly less blood loss in the Ten-Step method (400 vs. 80 mL) and the operation time was shorter (52.5 vs. 30.3 min) [11].

In a study in Croatia comparing 46 operations carried out using the Heaney method compared to 20 with the Ten-Step Vaginal Hysterectomy method, all with prolapsed uteri, the operation time with the Ten-Step method was significantly shorter (30 vs.45 min), and the needed time form analgesics shorter (30 vs. 40 h) [12] and the hospital stay was shorter (4.04 vs.3.32 days) [12].

In a study in Turkey comparing 24 operations carried out with the Heaney method compared to 25 with the Ten-Step Vaginal Hysterectomy method, the operation time with the Ten-Step method was shorter (97.92 vs. 50 min) [14].

The averages of age, BMI, number of pregnancies, number of live births, and pre- and post-operative hemoglobin levels were similar in both methods (*p* > 0.05). However, the patients who underwent the Ten-Step Vaginal Hysterectomy method had a significantly shorter operation time (*p* = 0.001), shorter hospital stay (*p* = 0.020) and shorter time of analgesics requirement (*p* = 0.006).

Despite current extensive use of endoscopy and robotics, vaginal hysterectomy should always be considered an option and should become part of each gynecological department’s arsenal. Studies comparing the “Ten-Step Vaginal Hysterectomy” to endoscopic hysterectomy should be encouraged and become the method of choice [15,16].

The limitations of this method are that it cannot be used for malignancies, severe abdominal adhesions, or very big uteri. Only surgeons with knowledge of anatomy should use it.

## 6. Conclusions

The “Ten-Step Vaginal Hysterectomy” is a valid method that should be highly considered when a hysterectomy is indicated. The prerequisites are the training of the surgeons and staff, knowledge of anatomy, and individual personal judgment of the surgeon if he or she is able to perform it. The benefits of this operation are short operation time, early mobility, low need for analgesics, and lack of scars. This method should receive its place again, as there are several basic principles that should remain with us despite the ongoing impressive surgical developments [17]. Due to its simplicity, the Ten-Step Vaginal Hysterectomy can be used globally, including in countries with low resources, where endoscopy or robotic surgery is not available.
